# Point-of-Care Transesophageal Echocardiography in Emergency and Intensive Care: An Evolving Imaging Modality

**DOI:** 10.3390/biomedicines13112680

**Published:** 2025-10-31

**Authors:** Debora Emanuela Torre, Carmelo Pirri

**Affiliations:** 1 Department of Cardiac Anesthesia and Intensive Care Unit, Cardiac Surgery, Ospedale dell’Angelo, Mestre, 30174 Venice, Italy; 2 Department of Neurosciences, Institute of Human Anatomy, University of Padova, 35121 Padova, Italy; carmelo.pirri@unipd.it

**Keywords:** transesophageal echocardiography, point-of-care ultrasound, emergency echocardiography, cardiac arrest, shock states, pulmonary embolism, focused cardiac ultrasound, artificial intelligence echocardiography

## Abstract

Transesophageal echocardiography (TEE) has long been established as a cornerstone imaging modality in cardiac surgery and perioperative medicine. In recent years, however, its role has expanded into emergency and intensive care settings, where rapid and accurate hemodynamic assessment is crucial for survival. Point-of-care TEE provides advantages over transthoracic echocardiography when acoustic windows are limited, particularly in mechanically ventilated or critically unstable patients, allowing continuous high-quality visualization of cardiac function, volume status, and great vessel pathology to guide immediate therapeutic interventions. This narrative review examines the evolving role of TEE in acute settings, with emphasis on its application in shock, cardiac arrest, pulmonary embolism, tamponade, and its value in extracorporeal membrane oxygenation (ECMO) cannulation. Advances such as three-dimensional TEE, miniaturized probes, and the integration of artificial intelligence are also discussed, as potential drivers of innovation. While bridging technological progress with clinical practice, TEE emerges as a versatile tool in critical care. However, its broader adoption is still limited by probe availability, operator training, and institutional resources. Overcoming these barriers will be essential to translating technological advances into widespread practice.

## 1. Introduction

Transesophageal echocardiography (TEE) has long been regarded as an indispensable tool in cardiac surgery and perioperative care, delivering high-fidelity visualizations of cardiac chambers, valves, and great vessels. More recently, its role has undergone a paradigmatic shift: point-of-care TEE is now emerging as a pivotal imaging modality in emergency and critical care settings, where rapid and precise hemodynamic evaluation can be decisive for patient survival [1]. In critically unstable or mechanically ventilated patients, where transthoracic acoustic windows may be unachievable, TEE offers indisputable advantages over transthoracic echocardiography (TTE), yielding superior image quality and enabling continuous evaluation even during cardiopulmonary resuscitation (CPR) [2,3]. This technological advance enhances the clinician’s ability to assess cardiac function, preload, afterload, and pump performance in real time. Particularly in shock states, cardiac arrest, pulmonary embolism, and pericardial tamponade, point-of-care TEE allows rapid identification of reversible causes and informs immediate clinical decisions. For instance, intra-arrest TEE can detect tamponade, intracardiac thrombi, and aortic dissection, and visualize real-time cardiac imaging without interrupting chest compressions [4,5]. Simultaneously, TEE is proving invaluable in facilitating extracorporeal membrane oxygenation (ECMO). It streamlines cannulation, confirms correct cannula positioning, and assists in volume management and ventricular unloading, particularly beneficial in cardiopulmonary failure contexts [6].

Beyond clinical applications, technological advances are expanding TEE’s potential. Three-dimensional (3D) TEE contributes richer spatial details, especially in complex anatomical or surgical scenarios [7]. Miniaturized and even disposable probes enhance safety and practical deployment in intensive care settings [8]. Moreover, early-stage integration of artificial intelligence enables automated quantification of left ventricular function, allowing consistent and rapid analysis [9]. Taken together, these developments underscore TEE’s dual identity as both a mature diagnostic tool and a rapidly evolving technology, blending innovation with immediate clinical applicability. This article is structured as a narrative review, aiming to qualitatively synthesize the available literature rather than perform a meta-analysis. Given the heterogeneity of study designs, patient populations, and clinical contexts, a narrative approach allows for a broader and more integrative understanding of the evolving role of point-of-care TEE in acute care medicine.

## 2. Materials and Methods

This work is designed as a narrative review aiming to provide a qualitative synthesis of the available evidence on point-of-care transesophageal echocardiography in emergency and intensive care medicine. The narrative format was chosen to accommodate the wide methodological and clinical heterogeneity of published studies, precluding formal meta-analytic synthesis but enabling a contextual appraisal of both clinical and technological dimensions. A comprehensive literature search was conducted across PubMed/MEDLINE, Scopus, Embase, the Cochrane Library, and ClinicalTrials.gov to identify relevant studies published between January 1990 and September 2025. The search combined controlled vocabulary (MeSH/Emtree) and free-text terms including “transesophageal echocardiography”, “Intensive care”, “Emergency”. No language restrictions were applied. Reference lists of key articles and review papers were also manually screened to identify additional eligible studies. In addition, studies that were judged relevant and informative based on citations within the bibliographies of the screened articles were also included, to ensure comprehensive coverage of the available evidence.

**Figure 1:**
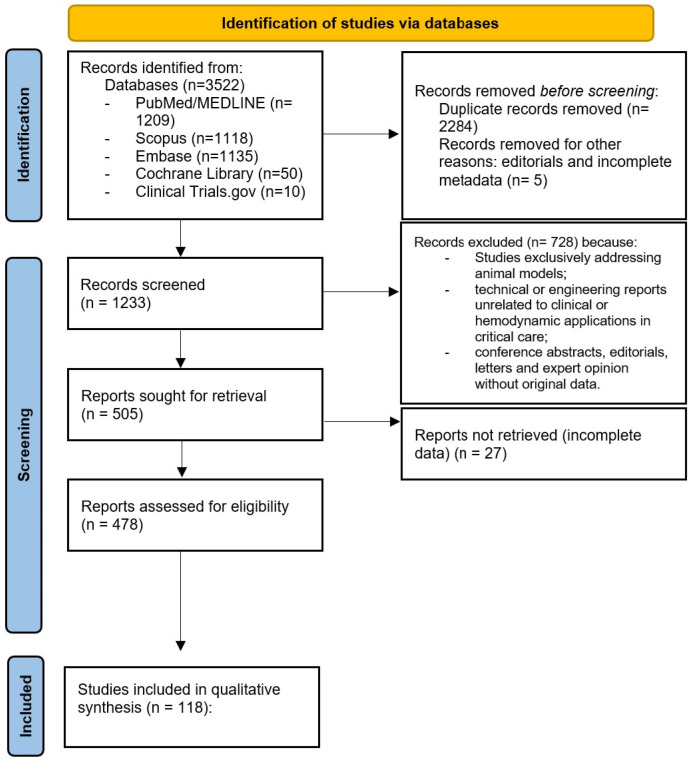
PRISMA-style flow diagram of the literature selection process.

Eligible articles included original clinical investigations, observational cohorts, case series, case reports, and review articles addressing the use of transesophageal echocardiography in acute or critical care settings, including its application in shock, cardiac arrest, pulmonary embolism, pericardial tamponade, and extracorporeal membrane oxygenation (ECMO). Studies were also included if they explored technological innovations, such as three-dimensional imaging, miniaturized probes, or artificial intelligence, relevant to emergency and intensive care practice. Exclusion criteria were as follows: studies exclusively addressing animal models; technical or engineering reports unrelated to clinical or hemodynamic applications in critical care; and conference abstracts, editorials, letters, and expert opinions without original data. Given the heterogeneity in study design, patient population, imaging protocols, and reported outcomes, no formal meta-analysis or statistical heterogeneity testing was performed. The evidence was therefore synthesized qualitatively, emphasizing methodological rigor, reproducibility, and clinical relevance. Quantitative parameters were incorporated where available, and limitations related to operator dependency and technological variability were explicitly acknowledged. Although this work was designed as a narrative review rather than a systematic one, a PRISMA-style flow diagram was nevertheless included to enhance transparency and reproducibility in the literature selection process, outlining the number of records identified, screened, assessed for eligibility, and included in the final quantitative synthesis (Figure 1).

## 3. Relevant Sections

### 3.1. Introduction to the Physical Principles of Transesophageal Echocardiography

TEE relies on the propagation of high-frequency ultrasound waves, which are partially reflected at tissue interfaces with different acoustic impedances. The esophageal route places the transducer in close proximity to the heart, avoiding interference from the chest wall, lungs, and adipose tissue. This anatomical advantage provides high spatial resolution and stable acoustic windows, particularly for posterior cardiac structures such as the left atrium, interatrial septum, and thoracic aorta. By integrating two-dimensional, Doppler, and three-dimensional modalities, TEE offers a comprehensive and dynamic evaluation of cardiac anatomy and hemodynamics [10].

### 3.2. Clinical Advantages in the Critical Care and Emergency Setting

**Figure 2:**
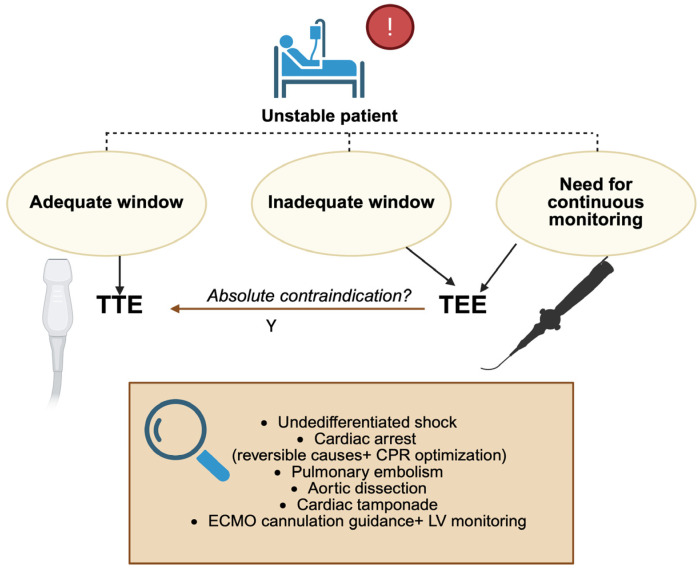
Decision algorithm: when to use TEE in the ED and ICU; TTE: transthoracic echocardiography; TEE: transesophageal echocardiography; LV: left ventricle; CPR: cardiopulmonary resuscitation; ECMO: extracorporeal membrane oxygenation. Created in BioRender. Pirri, C. (2025) https://BioRender.com/51zeqyz.

The comparative performance of transthoracic echocardiography (TTE) and transesophageal echocardiography (TEE) becomes particularly relevant in critically ill and unstable patients [11]. Although TTE is generally the first-line imaging modality due to its portability, non-invasiveness, and ease of application, its diagnostic accuracy is frequently compromised by poor acoustic windows in patients with mechanical ventilation, thoracic dressings, obesity, or postoperative chest wall changes. Under these conditions, TEE provides a decisive advantage: by positioning the transducer within the esophagus, it bypasses pulmonary parenchyma and bony structures, consistently delivering high-resolution images that remain reproducible irrespective of ventilatory status or body habitus [12,13] (Figure 2), (Table 1).

**Figure 3:**
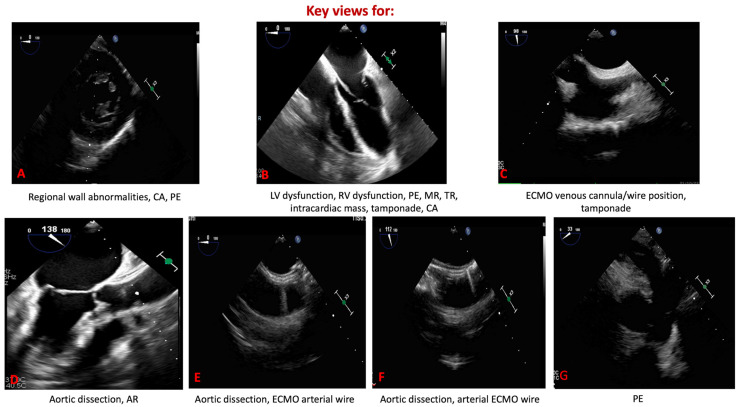
Key transesophageal echocardiographic views in emergency and critical care. (**A**) Trans-gastric mid-papillary short axis. (**B**) Mid-esophageal four-chamber view. (**C**) Mid-esophageal bicaval view. (**D**) Mid-esophageal aortic valve axis view. (**E**) Mid-esophageal short-axis aortic view. (**F**) Mid-esophageal aortic long axis view. (**G**) Mid-esophageal short-axis view of the ascending aorta. CA: cardiac arrest; PE: pulmonary embolism; LV: left ventricle; RV: right ventricle; MR: mitral regurgitation; TR: tricuspid regurgitation; ECMO: extracorporeal membrane oxygenation; AR: aortic regurgitation.

In the intensive care unit (ICU), this superiority translates into more reliable assessment of ventricular filling, contractile performance, and outflow hemodynamics in patients with undifferentiated or evolving shock. Unlike TTE, which may yield equivocal findings, TEE enables continuous or repeated monitoring and thereby guides titration of fluids, vasoactive agents, or mechanical circulatory support with greater precision [14]. Its value is especially evident during ECMO, where TEE not only facilitates accurate cannula positioning but also permits real-time evaluation of ventricular unloading and early detection of complications that might be missed with TTE [15]. The emergency department (ED) represents another domain in which the distinction between TEE and TTE is critical [16]. TTE is often attempted first in cardiac arrest or shock, but its utility is limited when image quality is compromised or when chest compressions must not be interrupted. Focused or resuscitative TEE provides uninterrupted, high-fidelity visualization throughout resuscitation, enabling immediate identification of reversible causes such as cardiac tamponade, pulmonary embolism, or acute aortic syndromes [17,18]. Moreover, TEE has proven superior to TTE in differentiating true electromechanical dissociation from pseudo-pulseless electrical activity and in guiding chest compression placement to optimize cardiac output [19,20]. Taken together, these attributes underscore the complementary but unequal roles of the two modalities: TTE remains an accessible, first-line tool, whereas TEE emerges as the most reliable option whenever immediate, high-quality cardiac imaging is required for life-saving decision-making in both the ICU and ED (Figure 3).

### 3.3. Reproducibility and Methodological Reliability of Quantitative TEE/TTE

Although echocardiography is inherently operator-dependent, reproducibility remains the cornerstone of its clinical reliability, particularly in unstable and mechanically ventilated patients. Early intraoperative TEE studies established the upper benchmark for quantitative consistency, reporting correlation coefficients up to r = 0.97 for repeated measurement of left ventricular function and less than 10% of segmental wall-motion discrepancies between observers (Deutsch et al. 1993) [21]. More recent investigations have confirmed that quantitative indices maintain clinically acceptable reproducibility even in technically demanding critical care environments (Table 2). In ventilated ICU patients with septic shock, De Geer et al. [22] reported an ICC of approximately 0.87 for LVEF, with a corresponding Pearson’s r = 0.78. These findings demonstrate that, when measured by trained intensivists, left ventricular systolic function can be assessed with high precision despite acoustic and hemodynamic challenges. Similarly, the left ventricular outflow tract velocity-time integral (LVOT VTI), a surrogate for stroke volume, has shown robust reproducibility across ICU operators. In a multicenter prospective study, Villavicencio et al. [23] found excellent intra-observer and good inter-observer agreement for LVOT diameters, VTI, and CO measurements performed by critical care physicians. Such a result reinforces the reliability of serial hemodynamic monitoring using bedside echocardiography. For right ventricular longitudinal function, both emergency and perioperative data demonstrate strong inter-observer reliability. In an emergency department study, Daley et al. [24] reported high agreement between physicians for TAPSE measurement and visual estimation, while intraoperative TEE investigations by Korshin et al. [25] confirmed that TAPSE can be quantified feasibly (>90%) and with good concordance compared with transthoracic acquisition. Taken together, these studies indicate that, even under the acoustic, ventilatory, and logistical constraints of critical care, TEE and focused TTE yield reproducibility metrics (ICC 0.85–0.95), (CV < 15%) comparable to those achieved in elective surgical settings. This body of evidence supports the methodological robustness of quantitative echocardiography for serial hemodynamic monitoring and decision-making in the critically ill and emergency department.

### 3.4. The Role of TEE in Shock States

**Figure 4:**
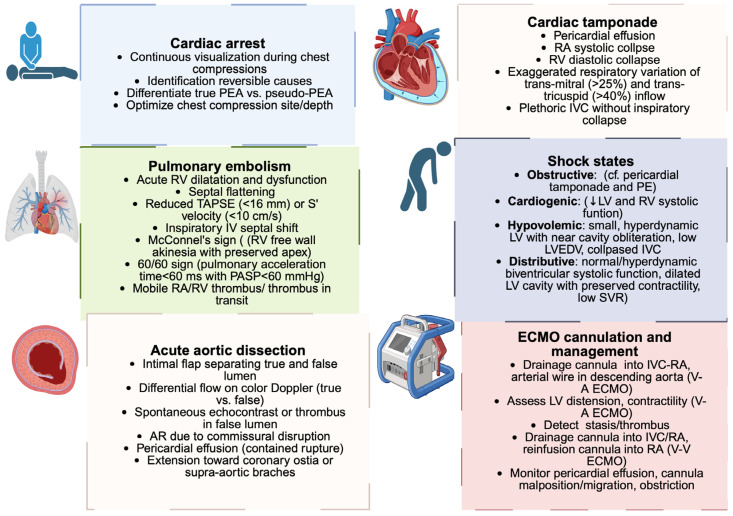
Echocardiographic findings in acute cardiovascular emergencies. Schematic overview of the main clinical conditions in which TEE plays a role: cardiac tamponade, pulmonary embolism, acute aortic dissection, cardiac arrest, ECMO cannulation, and the differentiation of shock states. Each box highlights the key echocardiographic features that support rapid diagnosis in an acute care setting. PEA: pulseless electrical activity; RA: right atrium; RV: right ventricle; IVC: inferior vena cava; PE: pulmonary embolism; LV: left ventricle; LVEDV: left ventricular end-diastolic volume; SVR: systolic vascular resistance; PASP: pulmonary arterial systolic pressure; TAPSE: tricuspid annular plane systolic excursion; AR: aortic regurgitation; ECMO: extracorporeal membrane oxygenation; V-A: veno-arterial; V-V: veno-venous. Created in BioRender. Pirri, C. (2025) https://BioRender.com/o2c04su.

In shock, where minutes matter, TEE turns hemodynamic assessment into a continuous, physiology-driven appraisal that remains reliable even when transthoracic windows fail [14] (Figure 4).

From the mid-esophageal and trans-gastric views, TEE depicts biventricular mechanics with a granularity that supports immediate decisions: left ventricular systolic performance can be gauged visually and by quantification (e.g., biplane Simpson), interpreting left ventricular ejection fraction (LVEF) in the context of established chamber quantification standards (normal values > 52% in men and >54% in women, according to ASE/EACVI guidelines) while integrating regional wall motion analysis to distinguish primary ischemic cardiogenic shock from global pump failure [26,27].

However, it is important to recognize that the acquisition of the optimal biplane views required for accurate LVEF measurement by the “modified” Simpson method can be challenging in unstable, mechanically ventilated patients, the very population for which TEE is typically deployed in ICU and emergency settings. Technical difficulties, suboptimal image quality, and patient instability often limit the feasibility and accuracy of quantitative biplane assessment in these settings. This limitation underscores the utility of complementary approaches such as the visual estimation of LVEF, which has been shown to have acceptable accuracy and a strong correlation (r = 0.818) with Simpson-derived LVEF even when performed by clinicians with limited echocardiographic experience. Visual estimation offers a rapid, practical assessment that can reliably guide urgent clinical decision-making when quantitative methods may be delayed or technically unfeasible [28].

Right-sided function, often the fulcrum in obstructive phenotypes, is captured with measurements such as tricuspid annular plane systolic excursion (TAPSE) and tissue-Doppler systolic velocity, where TAPSE below ~16 mm, S’~9.5–10 cm/s, or right ventricular fractional area chance (RVFAC) under ~35% coherently signal RV impairment and align with the visual cues of septal dyskinesis and RV/LV area mismatch [29].

Preload and filling pressures are inferred dynamically by coupling chamber sizes with Doppler patterns: trans-mitral inflow and tissue-Doppler indices (e.g., an elevated E/e’ consistent with raised left-sided filling pressures) help separate distributive states with high output physiology from cardiogenic profiles with impaired compliance.

For output and afterload assessment, serial measurement of the left ventricular outflow tract velocity time integral (LVOT VTI) provides a reliable surrogate for stroke volume [30].

In mechanically ventilated or critically ill patients, an increase in LVOT VTI ≥ 10–15% following a preload challenge, such as a passive leg raise or mini fluid bolus, is generally considered indicative of true fluid responsiveness [31,32]. Variations below this threshold usually reflect measurement variability of preload-insensitive cardiac performance. Accordingly, dynamic monitoring of LVOT VTI allows bedside discrimination between fluid responders and helps titrate volume and vasopressor therapy more safely. Crucially, the same examination shortens the path to a causal diagnosis: TEE reveals obstructive lesions within seconds, tamponade with diastolic right-sided collapse, acute RV pressure overload in pulmonary embolism, torrential valvular regurgitation from leaflet failure or proximal aortic dissection, so that therapy pivots from generic resuscitation to targeted intervention without breaking cadence at the bedside [33,34]. TEE allows precise, dynamic guidance of hemodynamic optimization, surpassing static pressure-based parameters and providing direct visualization of ventricular preload, contractility, and afterload. In a pivotal study by Perrino et al. [35], involving 33 patients, the cardiac output derived from multiplane TEE showed a mean bias of 0.01 L/min, a standard deviation of 0.56 L/min, and a correlation coefficient of r = 0.98 compared with pulmonary artery catheter (PAC)-derived thermodilution values, correctly tracking 97% of serial changes with only a modest 14% underestimation of their magnitude. These results correspond to a percentage error of approximately 18–25%, which lies within the interchangeability threshold proposed by Critchley and Critchley [36]. Therefore, TEE provides a less invasive yet quantitatively comparable alternative for real-time hemodynamic management in unstable patients, as demonstrated by Dabaghi et al. [37].

In summary, by fusing LVEF, RV indices, Doppler-derived filling pressures, and stroke volume surrogates into a single uninterrupted dataset, TEE upgrades shock evaluation from interference to direct visualization, and it does so in the very patients in whom TTE is most likely to be equivocal.

### 3.5. TEE in Pulmonary Embolism and Obstructive Shock

Pulmonary embolism (PE) represents one of the most challenging diagnoses in emergency and critical care, often manifesting as sudden cardiovascular collapse. In this context, TEE provides a decisive diagnostic advantage by directly visualizing the right heart and, in some cases, the embolic material itself [38]. Unlike TTE, which is frequently hampered by suboptimal windows in mechanically ventilated or peri-arrest patients, TEE offers high-resolution images of the right atrium, right ventricle, and main pulmonary arteries, enabling rapid confirmation of obstructive physiology at the bedside. TEE delineates acute right ventricular dysfunction through enlargement of the right ventricle, septal flattening, and impaired contractility, often accompanied by a depressed TAPSE (<16 mm) or reduced tissue Doppler S’ velocity (<10 cm/s). The identification of interventricular dependence, manifested by septal shift toward the left ventricle during inspiration, further corroborates the diagnosis of acute right ventricular pressure overload [39,40]. In massive PE, TEE may reveal mobile thrombi in transit across the right atrium or ventricle and even embolic obstruction at the pulmonary artery bifurcation, findings that carry immediate therapeutic implications [41]. Two echocardiographic signs are particularly noteworthy: the McConnell sign, characterized by akinesia or hypokinesia of the RV free wall with preserved apical contractility, and the 60/60 sign, defined by a pulmonary acceleration time < 60 ms in the presence of an estimated pulmonary artery systolic pressure < 60 m Hg. However, both signs lack specificity and must be interpreted in the clinical context [42,43]. Beyond diagnosis, TEE refines management in hemodynamically unstable patients with suspected PE. Its ability to distinguish obstructive shock from cardiogenic or distributive etiologies shortens time to reperfusion strategies such as thrombolysis, catheter-directed therapy, or surgical embolectomy. By integrating structural visualization, functional indices, and causal identification, TEE emerges as an indispensable modality in the evaluation of PE presenting as obstructive shock, particularly in scenarios where rapid differentiation determines survival [44]. Although TEE provides immediate hemodynamic and anatomical insights in massive PE, current international guidelines reaffirm that computed tomography pulmonary angiography (CTPA) remains the diagnostic gold standard [45,46]. In hemodynamically unstable patients, however, TEE serves as a crucial bedside help, bridging the gap between diagnostic uncertainty and therapeutic action. Beyond diagnostic accuracy, it is important to consider safety-related differences between TEE and CTPA. CTPA exposes patients to a mean effective radiation dose of approximately 3–6 mSv, with additional risk of contrast-induced nephropathy due to the use of 60–100 mL of iodinated contrast. In contrast, TEE involves no ionizing radiation and no nephrotoxic contrast agent, making it particularly advantageous for critically ill, pregnant, or renally impaired patients or when repeated assessments are required at bedside. This distinction underscores the complementary safety and accessibility of TEE as a rapid diagnostic alternative in emergency settings [47,48].

### 3.6. TEE in Cardiac Arrest

Cardiac arrest represents one of the most challenging scenarios in acute care, where uninterrupted resuscitation and immediate etiologic clarification are paramount. While TTE is frequently attempted during advanced cardiac life support, its utility is often undermined by poor image quality and the need to interrupt chest compressions. By contrast, resuscitative transesophageal echocardiography (rTEE) provides continuous, high-fidelity cardiac imaging throughout cardiopulmonary resuscitation (CPR), thereby transforming the approach to intra-arrest evaluation. The diagnostic value of rTEE extends beyond mere visualization of cardiac activity [16]. Within seconds, TEE can identify potentially reversible causes of arrest: pericardial tamponade with diastolic collapse of right-sided chambers, acute right ventricular dilatation and pressure overload in massive pulmonary embolism, structural valvular lesions such as flail mitral leaflet or ruptured chordae leading to acute severe mitral regurgitation and cardiogenic collapse, or proximal aortic dissection. The capacity to make these diagnoses in real-time without interrupting compressions enables immediate transition from non-specific resuscitation to targeted interventions such as pericardiocentesis, thrombolysis, or emergent surgical repair [49]. Equally important is the contribution of TEE to monitoring and optimizing the mechanics of resuscitation itself. By visualizing the cardiac chambers during compressions, rTEE allows precise adjustments of hand placement and compression depth, ensuring that external compressions maximize left ventricular outflow [19,50]. In addition, TEE distinguishes true pulseless electrical activity (PEA), characterized by the complete absence of mechanical contraction, from pseudo-PEA, in which organized myocardial contraction persists despite the lack of a palpable pulse. This distinction carries crucial prognostic and therapeutic implications, influencing decisions about the continuation or escalation of resuscitative efforts [51,52]. Evidence from the emergency department and intensive care cohorts demonstrates that rTEE alters both diagnosis and management in the majority of cardiac arrest cases. In a recent single-center case series (n = 25), Kegel et al. reported diagnostic clarification and management modification in 76% of patients, although no confidence intervals were provided and the authors acknowledged the exploratory nature of their results [16]. These findings are consistent with other small observational cohorts reporting management impact rates between 30% and 70% [17,18,19,20]. Accordingly, these estimates should be regarded as indicative rather than definitive, pending confirmation from larger multicentric studies.

### 3.7. TEE in Cardiac Tamponade

Cardiac tamponade represents a prototypical reversible cause of hemodynamic collapse, where rapid recognition is essential to avert circulatory arrest. While transthoracic echocardiography is the conventional first-line tool, image acquisition may be profoundly limited in mechanically ventilated or postoperative patients. In such scenarios, TEE provides superior and continuous visualization of pericardial and cardiac structures, even in technically challenging settings [30]. The echocardiographic diagnosis of tamponade relies on identifying a pericardial effusion exerting hemodynamic compromise, which manifests as diastolic collapse of the right atrium and right ventricle due to the trans-pericardial pressure gradient. In TEE, these findings are best appreciated in mid-esophageal four-chamber and bicaval views, where right atrial systolic inversion and right ventricular diastolic indentation can be detected with high sensitivity. Complementary Doppler interrogation reveals characteristic flow abnormalities, including exaggerated respiratory variation in trans-mitral (>25%) and trans-tricuspid (>40%) inflow velocities, consistent with ventricular interdependence [53,54]. Unlike TTE, TEE enables reliable detection of loculated or posterior effusions, which are frequently overlooked but highly relevant in postoperative or critically ill patients. Thus, by combining structural and functional criteria, TEE not only secures the diagnosis of tamponade with precision but also provides real-time guidance for pericardiocentesis or surgical drainage when definitive intervention is required [2].

### 3.8. TEE in ECMO Cannulation and Management

The expanding use of ECMO in refractory cardiogenic shock and severe respiratory failure has positioned TEE as a cornerstone imaging modality for both veno-arterial (VA) and venovenous (VV) configurations. During V-A ECMO cannulation, TEE guides safe wire advancement and verifies the correct placement of arterial and venous cannulae. Visualization of the venous drainage cannula is best achieved in the mid-esophageal bicaval view, confirming its course into the cavo-atrial junction or right atrium and ensuring appropriate orientation within the caval veins. For the arterial cannula, typically advanced retrogradely from the femoral artery into the descending aorta, mid-esophageal long- and short-axis views of the descending aorta provide the most reliable confirmation of position and exclude vascular complications. Once support is initiated, TEE provides continuous surveillance of left ventricular distension, residual systolic performance, and the presence of intracardiac stasis or thrombus, informing the decision on adjunctive unloading strategies and anticoagulation management [55,56,57]. In the ICU, TEE assumes equal importance in V-V ECMO, where oxygen delivery critically depends on effective drainage and reinfusion. By directly visualizing the drainage cannula in the inferior vena cava and the return cannula entering the right atrium with its jet directed toward the tricuspid valve, TEE ensures optimal separation of inflow and outflow streams, minimizing recirculation. Mid-esophageal bicaval and four-chamber views are essential for verifying cannula placement, including dual-lumen positioning and orientation of the reinfusion jet toward the tricuspid valve, and allow adjustment under echocardiographic guidance when flows are suboptimal [58,59]. TEE also enables early recognition of complications such as cannula migration, cannula obstruction, atrial wall contact, or pericardial effusion, which may not be apparent from circuit parameters alone [60,61,62].

### 3.9. TEE in Aortic Dissection

Aortic dissection represents a life-threatening emergency in which timely diagnosis directly determines survival. Computed tomography angiography remains the reference standard in stable patients; however, in unstable individuals or in intraoperative and critical care settings, TEE provides rapid, bedside evaluation with a diagnostic accuracy exceeding 95% [63,64]. By positioning the probe in close proximity to the thoracic aorta, TEE delivers high-resolution images of the ascending aorta, arch, and descending thoracic segments, enabling direct visualization of the intimal flap, differentiation of true and false lumen, and assessment of flow dynamics using color Doppler. Specific findings include spontaneous echo contrast or thrombus within the false lumen, entry and re-entry tears, and variations in luminal expansion during the cardiac cycle [65,66]. Importantly, TEE can identify complications that influence management, such as involvement of the coronary ostia, pericardial effusion from contained rupture, or severe aortic regurgitation due to commissural disruption. In the perioperative context, TEE not only confirms the diagnosis but also guides surgical repair by delineating the proximal extent of the dissection and monitoring ventricular function during cardiopulmonary bypass [67,68]. Its portability and rapid acquisition make it particularly valuable when hemodynamic instability precludes transfer for cross-sectional imaging, thereby establishing TEE as an indispensable diagnostic and monitoring tool in the acute care pathway of aortic dissection.

### 3.10. Clinical Scenarios Where TEE Provides Incremental Value over TTE

Although transthoracic echocardiography (TTE) remains the first-line imaging modality in most critically ill patients, its diagnostic yield may be limited by poor acoustic windows, mechanical ventilation, or complex postoperative anatomy. In these circumstances, TEE provides incremental diagnostic and procedural value, offering high-resolution, real-time visualization of cardiac structures when TTE findings are inconclusive or insufficient [69].

- Prosthetic valve dysfunction represents one of the clearest scenarios where TEE surpasses TTE. In mechanical or bioprosthetic valves, acoustic shadowing frequently obscures leaflet motion and thrombus detection on TTE. TEE, by providing en face and longitudinal views, allows the detection of abnormal masses suggestive of thrombus, pannus, or vegetations. Although definitive differentiation among these entities may require multimodality imaging, TEE remains essential for helping to guide management decisions, including the choice between fibrinolysis (in cases of confirmed prosthetic valve thrombosis), urgent surgery, or conservative therapy, depending on the underlying lesion [70,71,72].
- Infective endocarditis remains a major field in which TEE is considered the gold standard. It provides superior sensitivity and specificity for detecting vegetations, periannular abscesses, pseudoaneurysms, and prosthetic valve involvement, especially in patients with inconclusive TTE or systemic embolization. In this setting, early TEE can change the therapeutic approach by expediting antimicrobial therapy or surgical referral in high-risk cases [73].
- In post-myocardial infarction or post-interventional mechanical complications, such as ventricular septal rupture or free wall rupture, TEE enables immediate diagnostic clarification and dynamic hemodynamic assessment at the bedside. Its continuous monitoring capability is particularly valuable during hemodynamic instability or extracorporeal support, allowing real-time evaluation of shunt direction, pericardial effusion, and ventricular interdependence [74].
- In anomalous coronary artery origin, particularly anomalous left or right coronary arteries arising from the opposite sinus (anomalous aortic origin of the left coronary artery, AAOCA-L, or anomalous aortic origin of the right coronary artery, AAOCA-R), TEE can complement CT angiography or coronary angiography by providing rapid confirmation of the coronary course. This is especially relevant in young patients presenting with exertional syncope, ventricular arrhythmias, or cardiac arrest, in whom prompt recognition may be lifesaving [75].

Finally, in undifferentiated shock and ECMO cannulation, TEE remains invaluable when TTE is technically unfeasible.

### 3.11. Contraindications and Complications of TEE

Despite its established safety profile, TEE remains a semi-invasive procedure and should be performed with awareness of its contraindications and potential complications (Table 3). Absolute contraindications include esophageal perforation, active upper gastrointestinal bleeding, esophageal diverticulum, and known esophageal tumors, while relative contraindications encompass severe coagulopathy, esophageal varices, large hiatal hernia, and recent esophageal or gastric surgery [76]. In critically ill patients, these conditions must be carefully balanced against the diagnostic yield, as emergent scenarios may still justify the procedure under heightened precautions [77]. Reported complication rates are low, typically <1%, with most events being minor, such as transient sore throat, hoarseness, or minor mucosal trauma. More serious but rare complications include esophageal laceration or perforation, gastrointestinal bleeding, dental injury, and arrhythmias triggered by probe manipulation [78,79]. The overall risk increases with prolonged procedures, difficult probe insertion, and in patients with pre-existing esophageal pathology. Thus, meticulous patient selection, adherence to insertion protocols, and prompt recognition of warning signs are essential to maintaining the favorable risk-benefit profile of TEE in both perioperative and critical care practice [80].

### 3.12. Three-Dimensional TEE in Emergency and Critical Care

The advent of three-dimensional transesophageal echocardiography (3D-TEE) has expanded the diagnostic and procedural armamentarium of critical care echocardiography [81,82]. By providing volumetric datasets and anatomically realistic reconstructions, 3D-TEE overcomes many of the geometric assumptions inherent to two-dimensional imaging, thereby enhancing accuracy in chamber quantification, valve assessment, and spatial orientation [26,83]. This advantage has been robustly validated in numerous studies demonstrating superior accuracy and reproducibility of 3D volumetric measurement compared to 2D echocardiography, with validation against cardiac magnetic resonance imaging (CMR) as the gold standard [84].

For example, Jenkins et al. showed that real-time 3D-TEE achieves significantly lower inter- and intra-observer variability, with a coefficient of variation reduction from approximately 10–15% in 2D to 5–7% in 3D, thereby improving reliability in clinical decision-making [85].

Moreover, Mor-Avi et al. [86] demonstrated an excellent correlation (r = 0.95) between 3D-TEE and CMR for ventricular volumes (compared with r = 0.79 for 2D-TEE), with minimal systematic bias and less volume underestimation (2D-TEE bias 39% vs. 3D-TEE bias 3%). In addition, 3D-TEE reduced inter-observer variability from 37% to 7% and intra-observer variability from 19% to 8%.

In the emergency department and ICU, these advantages translate into more precise delineation of complex pathologies such as acute valvular disruption, prosthetic valve thrombosis, and intracardiac thrombi, all of which may precipitate shock or cardiac arrest [87]. Real-time 3D datasets also facilitate periprocedural guidance: in V-A ECMO, 3D-TEE supports cannula positioning by offering en face visualization of the atrial septum and caval inflows [88,89] and by improving spatial orientation during cannulation and guidewire positioning, thereby enhancing safety and precision through real-time visualization of the cannula trajectory and tip. Importantly, 3D-TEE has demonstrated value in refining the hemodynamic assessment of critically ill patients, allowing direct volumetric estimation of ventricular function and dynamic evaluation of right ventricular geometry, parameters frequently underestimated with conventional 2D techniques [26]. Although limited by availability, probe size, and the need for advanced expertise, 3D-TEE is emerging as an indispensable complement to conventional TEE, bridging anatomic precision with real-time hemodynamic guidance in the acute care setting.

### 3.13. Miniaturized and Disposable TEE Probes

Recent advances in probe technology have extended the feasibility of TEE beyond traditional perioperative use. Miniaturized probes, characterized by reduced shaft diameter and enhanced flexibility, allow safe deployment in patients previously considered unsuitable for standard TEE, including those with limited esophageal tolerance or in pediatric and neonatal populations. Their smaller footprint also facilitates prolonged bedside monitoring in the ICU, where conventional probes may be poorly tolerated [90,91,92]. Parallel to miniaturization, the development of disposable TEE probes has addressed critical concerns of infection control and equipment availability. Single-use designs mitigate the risk of cross-contamination, simplify workflow by eliminating the need for high-level disinfection, and ensure immediate access in emergency scenarios [8,93,94,95].

While image resolution may be modestly lower than that of high-end reusable systems, disposable and miniaturized TEE probes have shown acceptable diagnostic performance in preliminary clinical studies, supporting their use for rapid hemodynamic assessment, procedural guidance, and detection of life-threatening pathologies [92,93]. In a study including 53 examinations, Hlaing et al. reported that 77% of miniaturized TEE probes studies provided management-relevant information, with usefulness reaching 92% in patients without mechanical circulatory support (MCS) and 64% in those with MCS devices, yielding an odds ratio of 0.156 (95% CI 0.015–0.988, *p* = 0.022) for reduced usefulness in the MCS group. Only 15% of examinations were rated as having poor image quality.

Together, these innovations are broadening the reach of TEE, embedding it as a practical, safe, and immediately deployable modality in emergency and critical care medicine.

### 3.14. Artificial Intelligence and Image Processing with TEE

The integration of artificial intelligence (AI) into TEE represents a rapidly developing field in critical care imaging. Deep learning models trained on large echocardiographic datasets have already demonstrated the capability to automatically detect anatomic landmarks, segment cardiac chambers, and quantify ventricular function with accuracy approaching that of expert interpretation [96,97]. In a validation study including 10,030 echocardiograms, automated LVEF prediction achieved a Pearson’s r correlation coefficient of 0.83 compared with expert analysis (*p* < 0.001) and an AUC of 0.98 (95% CI 0.97–0.99) for identifying patients with reduced ejection fraction. External validation in an independent cohort of 200 echocardiograms confirmed robust performance with an AUC of 0.90 (95% CI 0.88–0.91). These algorithms substantially reduce inter-observer variability and analysis time while maintaining clinically acceptable accuracy in ventricular quantification [98].

In a pediatric cohort on ECMO support, a deep-learning algorithm (PEQ-Net) was shown to provide automatic assessment of LVEF with excellent agreement to expert manual measurements (ICC 0.983, 95% CI 0.977–0.987) and improved performance compared to junior echocardiographers, thus facilitating more accurate bedside quantification of ventricular function in the critical care setting [99].

Nonetheless, AI-assisted echocardiography remains in the early validation phase for acute and critical care applications, where motion artifacts, arrhythmias, and suboptimal acoustic windows still challenge automated analysis [100]. Accordingly, current evidence supports AI as a complementary tool that enhances reproducibility and efficiency in image interpretation, rather than a fully autonomous diagnostic modality [101].

### 3.15. Cost-Effectiveness and Practical Limitations

From an economic standpoint, the cost-effectiveness of TEE has been investigated across several clinical settings. In patients with atrial fibrillation, a decision-analytic model showed that TEE-guided early cardioversion was a dominant strategy, that is, both less costly and at least equally effective, compared with conventional delayed cardioversion, with an average cost of USD 2774 vs. 3070–3106 and a QALY gain of 8.49 vs. 8.48 [102].

Similarly, in catheter-related Staphylococcus aureus bacteremia, using TEE to tailor antibiotic duration yielded an incremental cost-effectiveness ratio of USD 4938 per QALY gained, indicating favorable economic value [103].

For comparison, the PAC-Man randomized trial evaluated the cost-effectiveness of pulmonary artery catheter (PAC) monitoring in ICU patients and found no survival benefit and higher costs for routine PAC use, with an estimated cost of approximately GBP 2985 per QALY gained when the device was withdrawn from standard management [104].

Collectively, these studies demonstrate that while TEE has shown favorable cost-effectiveness in defined diagnostic scenarios, comprehensive economic evaluations in the acute and critical care environment remain limited. Future research should aim to directly compare TEE-based versus PAC-based management strategies in terms of cost, clinical outcomes, and resource utilization.

Beyond cost considerations, several practical barriers currently limit the widespread adoption of point-of-care TEE in emergency and intensive care settings. These include equipment availability, operator training, and infection control measures.

The cost of dedicated TEE probes and their limited number in non-tertiary hospitals often restricts routine access. Implementing shared resource programs or centralized sterilization facilities may help mitigate such constraints, particularly in high-volume critical care units.

Training remains a critical factor for safe and effective use. Structured competency-based curricula, supervised hands-on courses, and certification pathways can enhance operator proficiency while ensuring patient safety. Simulation-based training may also accelerate learning curves, especially for emergency physicians and intensivists newly approaching TEE [105].

Infection control represents another key limitation. The use of high-level disinfection protocols, dedicated probe covers, or disposable single-use probes is essential to minimize cross-contamination. Recent advances in probe design and automated disinfection systems have improved feasibility and turnaround times, making TEE increasingly compatible with routine ICU workflows [106,107].

From an implementation standpoint, integrating point-of-care TEE into critical care practice should follow a stepwise approach: define clinical indications (e.g., undifferentiated shock, cardiac arrest, ECMO cannulation); ensure 24/7 probe accessibility; establish credentialing and revalidation standards; and develop interdepartmental collaboration between anesthesiology, cardiology, and critical care teams.

Collectively, these technological and organizational strategies outline a pragmatic and sustainable pathway for the broader clinical integration of bedside TEE.

### 3.16. Comparative and Critical Appraisal of Contemporary TEE Techniques

A comparative perspective on contemporary TEE techniques reveals complementary strengths and inherent limitations that shape their use in critical care. Conventional multiplane TEE remains the reference standard for high-resolution diagnostic imaging and Doppler quantification, yet it requires advanced expertise and patient sedation [2].

Real-time 3D-TEE enhances spatial orientation and volumetric accuracy, particularly during ECMO cannulation or complex valvular interventions, though its use is still constrained by probe availability and the need for advanced hardware and software processing [7].

Miniaturized and disposable hemodynamic TEE probes, while offering lower image resolution and limited Doppler capability, provide unmatched practicality for prolonged bedside monitoring in the ICU [8].

Artificial intelligence TEE interpretations are emerging as a transformative adjunct that reduces inter-observer variability and accelerates quantitative analysis, though expert validation remains essential [108].

A pragmatic integration of these modalities, guided by patient condition, operator proficiency, and institutional resources, represents the most balanced strategy toward precision hemodynamic monitoring in critical care.

## 4. Discussion

TEE has undergone a profound transformation, evolving from a perioperative monitoring tool into a frontline modality for acute cardiovascular care. Its clinical impact extends well beyond the operating room: in the emergency department and ICU, where rapid and accurate hemodynamic emergency is often decisive, TEE consistently outperforms transthoracic echocardiography whenever acoustic windows are compromised. By providing high-resolution, real-time imaging without interruption of resuscitative efforts, it enables both immediate etiologic clarification and dynamic guidance of therapy [14,109]. Although several quantitative parameters and diagnostic yields were extracted from primary studies, this review did not aim to conduct a statistical aggregation or heterogeneity analysis. The available studies on point-of-care transesophageal echocardiography display marked variability in design, patient population, operator experience, and measurement protocols, which makes pooled statistical synthesis inappropriate. Accordingly, this work is intentionally structured as a narrative qualitative review, integrating evidence across diverse clinical and technological settings to identify consistent diagnostic and therapeutic trends rather than to produce meta-analytic estimates. This methodological approach aligns with current standards for narrative reviews, where clinical and technological heterogeneity preclude formal statistical pooling but provide valuable contextual understanding.

However, the widespread implementation of TEE in the ED and ICU is still hampered by practical constraints. Unlike TTE, which is broadly available, TEE requires specialized equipment, trained personnel, and adherence to strict disinfection protocols. These resource-related limitations significantly restrict its accessibility outside cardiac surgery and high-volume centers, underscoring the need for structured training programs and institutional investment to facilitate broader clinical adoption.

What makes TEE particularly relevant in contemporary practice is its breadth of diagnostic application. In unstable patients, it offers rapid confirmation of life-threatening pathologies such as aortic dissection, cardiac tamponade, pulmonary thromboembolism, ventricular thrombi, atrial masses, and infective endocarditis [12,65,110,111,112]. This capacity positions TEE as an indispensable diagnostic bridge, complementing but not replacing the established gold standard. Equally important is the inherently multidisciplinary nature of TEE. Anesthesiologists, cardiologists, intensivists, and cardiac surgeons each bring a distinct interpretative lens: anesthesiologists emphasize perioperative stability and ECMO cannulation, cardiologists refine structural and valvular assessment, intensivists integrate findings into longitudinal shock management, while surgeons employ intraoperative imaging to guide complex repairs. When interpreted collaboratively, TEE findings gain a translational dimension, directly shaping individualized therapeutic strategies. Looking ahead, the integration of emerging technologies is set to redefine TEE’s scope. Three-dimensional imaging already enhances anatomical fidelity, while artificial intelligence promises automated quantification of ventricular function and reduction in inter-observer variability. Experimental applications of augmented reality and telemedicine point toward a future in which expertise can be projected across distance, enabling remote procedural guidance in both resource-rich and resource-limited environments [96,113,114]. Within such a paradigm, TEE is poised to align with the principles of precision medicine, offering real-time, patient-specific hemodynamic information that drives tailored interventions. In summary, the evolution of TEE reflects a dual trajectory: consolidation as a mature, indispensable imaging modality in critical illness and expansion as a platform for technological innovation and multidisciplinary integration. This duality ensures that TEE will continue to shape the future of acute cardiovascular care.

### Clinical Implications and Future Perspectives

The bedside use of TEE is progressively transforming critical care monitoring by enabling real-time visualization of cardiac function and rapid identification of reversible causes of hemodynamic instability. Its practical adoption requires structured training, probe accessibility, and robust infection control policies to ensure safe and sustainable integration into everyday ICU workflows. As miniaturized probes and AI-assisted quantification become increasingly available, these innovations are expected to enhance reproducibility and broaden access beyond tertiary centers. Future research should focus on outcome-based validation and standardized credentialing pathways to consolidate bedside TEE as an essential component of precision critical care. Looking ahead, intracardiac echocardiography (ICE) probes, used via the transesophageal route, are emerging as a promising, though still experimental, evolution of conventional TEE technology. This off-label approach has been explored in structural and interventional cardiology as a potential miniaturized alternative to standard TEE. Early experience by Mitchell-Heggs et al. confirmed the feasibility of using a conventional ICE probe through the esophageal route to guide percutaneous PFO closure. Adequate imaging was achieved in all patients, and diagnostic agreement between ICE and conventional TEE was excellent (r = 0.9), and no procedure-related complications occurred. The smaller probe diameter and superior tolerability may offer a practical advantage in hemodynamically unstable patients or in cases where prolonged TEE is poorly tolerated, reducing the need for general anesthesia or deep sedation [115].

Recent reports, including the DIONISO study, have demonstrated the safety and image quality of this approach during left atrial appendage occlusion (LAAO) procedures. Further research is warranted to define its diagnostic accuracy, safety profile, and potential applicability in critical care as a less invasive alternative to conventional TEE [116,117].

## 5. Conclusions

TEE has consolidated its role as an essential imaging modality in acute cardiovascular care, bridging diagnosis and therapy in scenarios where time and precision are critical. Its expanding integration with advanced technologies and multidisciplinary practice positions it as a driver of precision medicine, with the potential to reshape standards of care in emergency and intensive settings. However, its broader implementation is still constrained by the limited availability of probes, trained operators, and institutional resources, factors that currently restrict routine use outside specialized centers. Overcoming these barriers will be crucial to ensure that forthcoming technological advances can translate into wider clinical application.

## Figures and Tables

**Table 1:** Comparison of TTE vs. TEE in the ICU and ED.

Feature	TTE	TEE
Portability	High	Moderate
Invasiveness	Non-invasive	Semi-invasive
Imaging during CPR	Limited	Continuous, uninterrupted
Image quality	Window-dependent	Consistently high
Typical applications	First-line screening	Definitive diagnosis/monitoring

TTE: transthoracic echocardiography; TEE: transesophageal echocardiography; CPR: cardiopulmonary resuscitation; ICU: intensive care unit; ED: emergency department.

**Table 2:** Inter- and intra-observer reproducibility of quantitative echocardiographic parameters (TEE and TTE) across perioperative and critical care settings.

Parameter	Setting/Modality	Study	Variability (Statistical Index)	Main Evidence/Remarks
LVEF	TEE (intraoperative)	Deutsch HJ et al., 1993, Thorac Cardiovasc Surg [21]	r = 0.97 (inter-observer); segmental motion discrepancy ~9%	Early validation of intraoperative TEE reproducibility under controlled anesthetic conditions
LVEF	TTE (ICU, septic shock)	De Geer L et al., 2015, Cardiovasc ultrasound [22]	r = 0.78; ICC = 0.87 (95% CI 0.77–0.93)	Low variability even in mechanically ventilated patients
LV GLPS	TTE (ICU, ventilated patients)	De Geer L et al., 2015, Cardiovasc ultrasound [22]	k: 0.71 r: 0.84 ICC: 0.91 (95% CI 0.74–0.95)	Acceptable reproducibility for mitral annular tissue Doppler velocities
LVOT, VTI, CO	TTE (intensivist performing critical care echo)	Villavicencio C et al., 2019, Ultrasound J [23]	Intra-observer: excellent; inter-observer: good agreement for LVOT diameter, VTI, and CO (Fleiss index)	Validated reproducibility of VTI-based cardiac output among ICU physicians
TAPSE in PE	TTE (emergency department, physician-performed)	Daley J et al., 2017, Am J Emerg [24]	High inter-observer reliability (visual and quantitative TAPSE) k = 0.94 (95% CI, 0.87–0.98) ICC = 0.87 (95% CI, 0.79–0.93)	Emergency-physician-performed TAPSE proved accurate and reproducible in acute settings
TAPSE	TEE (perioperative cardiac surgery)	Korshin A et al., 2018, Int J Cardiovasc Imaging [25]	High feasibility (>90% and good agreement between TEE and TTE TAPSE)	Reliable quantification of RV function by TEE in surgical patients

TEE: transesophageal echocardiography; TTE: trans-thoracic echocardiography; ICC: intraclass correlation coefficient; r: Pearson’s r; k: Cohen’s k; LVEF: left ventricular ejection fraction; LV: left ventricle; RV: right ventricle; TAPSE: tricuspid annular plane systolic excursion; LVOT VTI: left ventricular outflow velocity time integral; GLPS: global longitudinal peak strain; ICU: intensive care unit.

**Table 3:** Contraindications to transesophageal echocardiography.

Absolute Contraindications	Relative Contraindications
Esophageal perforation	Esophageal varices
Active upper gastrointestinal bleeding	Severe coagulopathy
Esophageal tumor/malignancy	Large hiatal hernia
Esophageal diverticulum	Recent upper gastrointestinal or esophageal surgery
	History of esophageal strictures or dysphagia
	Cervical spine instability or severe arthritis (limiting neck extension)

## Data Availability

No new data were created or analyzed in this study. Data sharing is not applicable to this article.

## Abbreviations

The following abbreviations are used in this manuscript:

TTE	Transthoracic echocardiography
TEE	Transesophageal echocardiography
ECMO	Extracorporeal membrane oxygenation
PE	Pulmonary embolism
CA	Cardiac arrest
ME	Mid-esophageal
TG	Trans-gastric
